# Chronic type C3 distal humeral fracture associated with massive bone defects treated by open reduction and internal fixation with iliac crest autografts: a case report

**DOI:** 10.1186/s12891-021-04199-4

**Published:** 2021-04-07

**Authors:** Yejun Zha, Kehan Hua, Maoqi Gong, Xieyuan Jiang

**Affiliations:** Department of Orthopedic Trauma, Beijing Jishuitan Hospital, No.31 Xinjiekou East Street, Xicheng District, Beijing, 100035 China

**Keywords:** Distal humeral fracture, Chronic intercondylar fracture, Bone defect, Open reduction and internal fixation, Iliac crest autograft

## Abstract

**Background:**

Chronic intercondylar fractures of the distal humerus with massive bone defects and severe comminution in the metaphysis are rare and complex injuries that are challenging for surgeons to treat, as reconstructing the triangular structure of the distal humerus is difficult and may have a severe impact on functional outcomes, especially in young patients, for whom total elbow arthroplasty is usually not a suitable option due to significant impairment in upper limb strength. Here, we report a patient in such scenario who was young and active and was treated by structural iliac bone autografting and internal fixation.

**Case presentation:**

A 26-year-old male patient experienced a major car accident and was diagnosed with an open fracture (Gustilo-Anderson type IIIB) of the right distal humerus with massive bone defects and severe intra-articular involvement, without neurovascular injuries or other associated injuries. Surgical debridement, negative pressure vacuum sealing drainage, and immobilization by braces were initially performed, and the wound was closed after 15 days. When the wound had finally healed and the soft tissue was in good condition without infection or effusion 45 days later, this young and active patient was diagnosed with a chronic type C3 distal humeral fracture associated with massive bone defects at the supracondylar level in both columns and severe comminution at the trochlear groove. We performed surgical debridement and arthrolysis around the fracture site, and then, we successfully reconstructed the triangular structure of the distal humerus using structural iliac crest autografts in both columns as well as in the defective trochlear groove. Finally, internal fixation via a parallel double-plate configuration was performed. Over a follow-up period of 3 years, the patient achieved almost full recovery of range of motion and an excellent functional score, without minor or major postoperative complications.

**Conclusion:**

In this study, we proposed a surgical reconstruction strategy for complex chronic distal humeral fractures associated with massive bone defects and severe articular involvement in young and active patients using metaphyseal shortening and structural iliac crest bone autografting together with open reduction and internal fixation via a parallel configuration.

## Background

Intercondylar fractures of the distal humerus, which are relatively rare and complex, are often associated with severe soft tissue lesions and multiple fracture fragments [1]. Usually, open reduction and rigid internal fixation, as well as proper rehabilitation protocols, are performed as early as possible to achieve good clinical outcomes [2, 3]. However, for rare cases with compound fractures and massive bone defects as well as severe comminution, surgeons may only perform debridement, wound closure, and immobilization of the elbow by casting instead of one-stage open reduction and internal fixation to prevent infection. Therefore, these cases often exhibit delayed healing when the patients are transferred to trauma centers several months later. The absence of signs of bone healing, massive bone defects and severe displacement of the fracture fragments pose great challenges for orthopedic surgeons regarding the reconstruction of the distal humerus and may even deleteriously affect elbow function postoperatively [4, 5]. For elderly patients, total elbow arthroplasty (TEA) can be a safe and reliable choice in such scenarios [6]. However, for most young and active patients, TEA may significantly compromise the strength of the upper limbs, so we have to reconstruct the distal humerus by rigid internal fixation as much as we can. Here, we report a case of a chronic type C3 fracture of the distal humerus associated with severe bone defects that was treated by iliac bone autografting and internal fixation.

## Case presentation

A 26-year-old male patient experienced a car accident and was diagnosed with an open fracture (Gustilo-Anderson type IIIB) of the right distal humerus with massive bone defects and severe intra-articular involvement, without neurovascular injuries or other associated injuries. Within 24 h after the injury, he was treated by surgical debridement, negative pressure vacuum sealing drainage, and immobilization by casting in a local hospital. Due to severe contamination and a poor soft tissue condition, the wound was surgically debrided again and closed 15 days later. Two months after the initial operation, the wound had finally healed, and the soft tissue was in good condition, without infection or effusion. The CRP (C-reactive protein) and ESR (erythrocyte sedimentation rate) levels returned to normal, and the patient was transferred to our department for additional treatment.

**Fig. 1:**
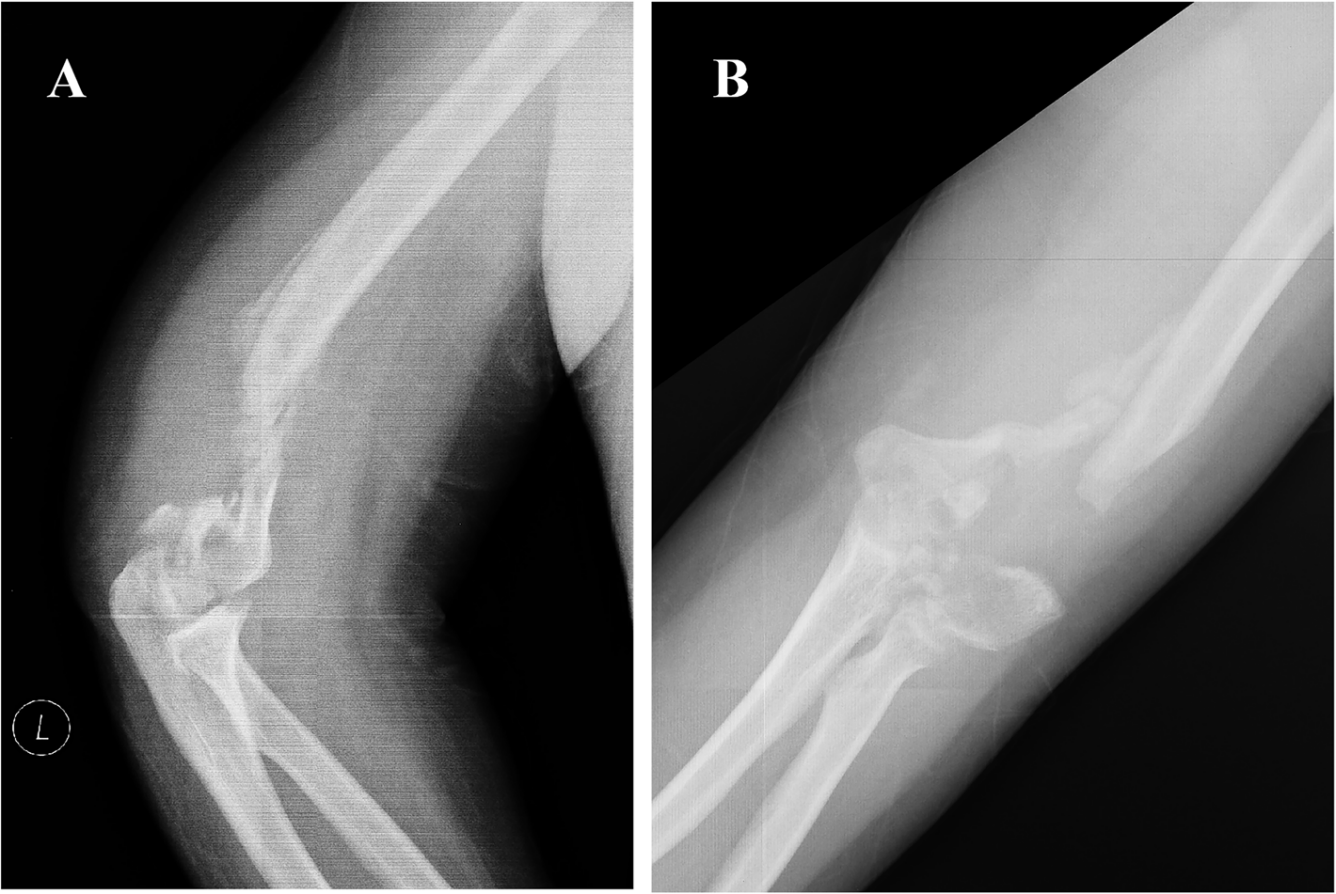
Preoperative lateral (**a**) and AP (**b**) view of X-rays showed massive bone defects at the supracondylar level

**Fig. 2:**
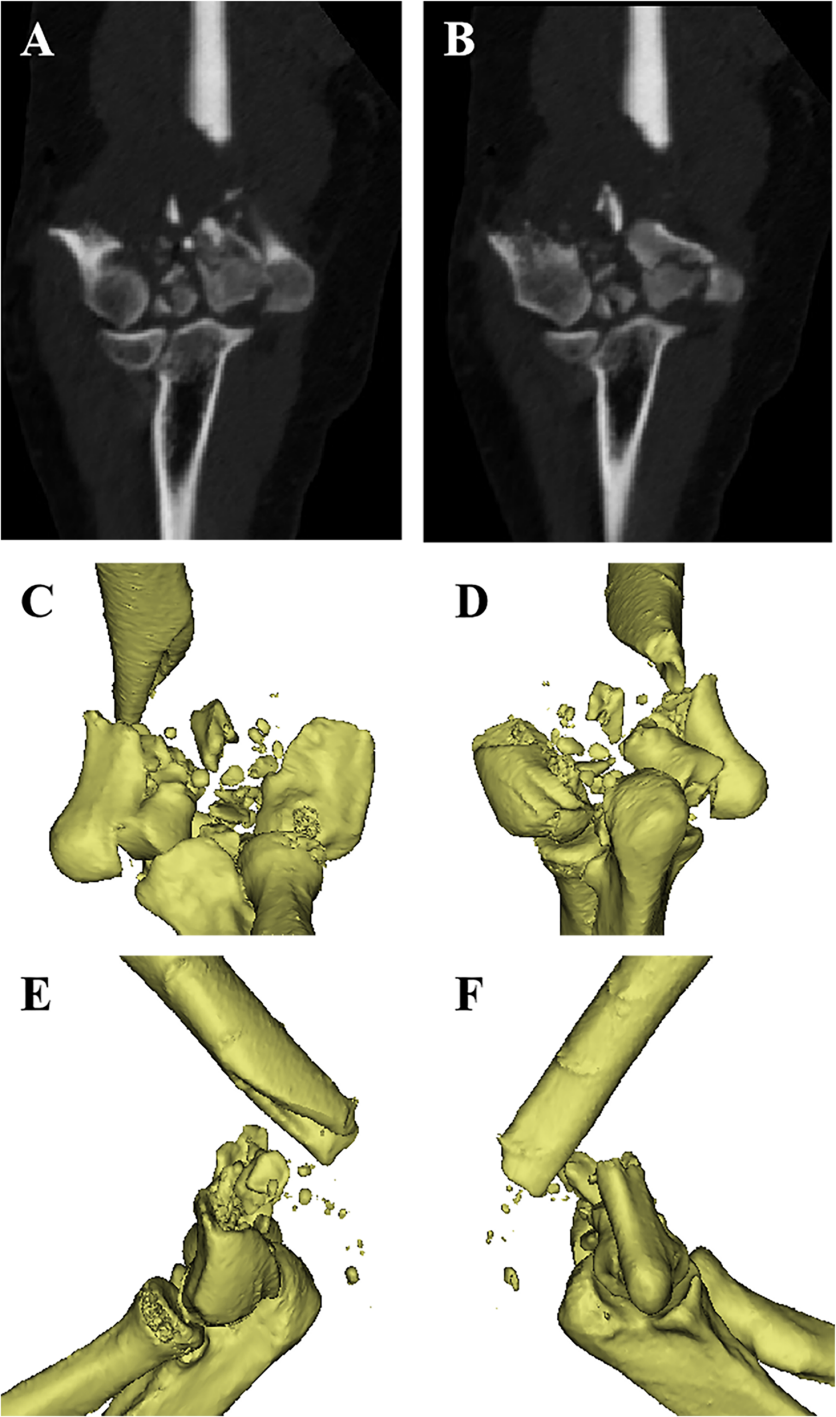
Preoperative CT scan (**a**, **b**) and 3D reconstruction (**c-f**) showed severe comminution of the articular surface and massive bone defects at supracondylar level in both columns

**Fig. 3:**
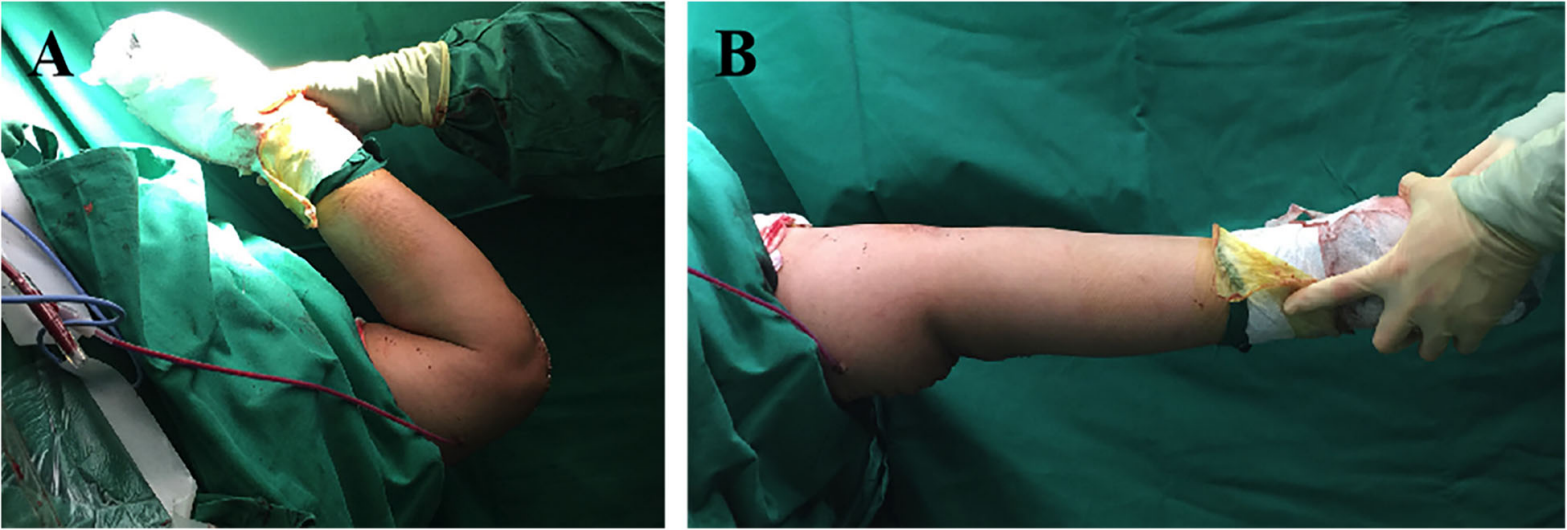
The flexion (**a**) and extension (**b**) function of the involved elbow after internal fixation before wound closure

The patient’s height was 175 cm, and his weight was 130 kg. The preoperative anteroposterior (AP) and lateral X-rays (see Fig. 1) and 3D-CT scans (see Fig. 2) of the right elbow joint showed massive bone defects at the supracondylar level as well as a comminuted articular surface. According to the Association for Osteosynthesis/Association for the Study of Internal Fixation (AO/ASIF) criteria, the fracture was classified as a type 13-C3 fracture [7]. The physical examination revealed pseudarthrosis at the fracture site, which made it much more difficult to reconstruct the distal humerus.

### Surgical procedure

After the induction of general anesthesia, the patient was placed in the supine position with elbow flexion and forearm crossing chest [8], and a longitudinal incision was made along the midline of the posterior aspect of the elbow and medially curved at the olecranon tip. The ulnar nerve was dissected carefully and protected by a rubber strip, and then, a V-shaped osteotomy was performed in the proximal olecranon. The proximal bone fragment and triceps muscle were flipped upward to expose the distal part of the humerus.

Then, we removed all the fibrous scar tissue as well as the anterior and posterior capsules to release the elbow. The dead bones and redundant calli were debrided until fresh bone was evidently revealed, and then the bone callus was kept for grafting. The original articular cartilage was preserved to the greatest extent possible, but the trochlear groove was too severely comminuted to be reconstructed. Therefore, the fracture fragments and adhesive fibrous tissue were removed to facilitate reconstruction.

The trochlear and capitellar articular surfaces of the distal humerus were aligned with the olecranon and radial head articular surfaces, respectively. Then, we measured the width of the trochlear groove defect, harvested a cylindrical autograft of an appropriate size and shape from the iliac crest, and inserted the graft into the defect to reconstruct the distal humerus. The cortical bone surface of the graft was directed towards the articular cavity but was located 2 mm proximal to the cartilage. We stabilized the distal fragments using K-wires (Kirschner wires) for temporary reduction. Then, the intercondylar fracture was converted to a supracondylar fracture of the distal humerus.

Next, the humeral shaft and both columns were reduced. First, the medullary canal was opened by a 3.5 mm diameter drill to promote fracture healing. The supracondylar bone defects were measured to be approximately 3 cm at the medial column and 5 cm at the lateral column. We performed shortening by 2 cm at the supracondylar level. Then, 2 pieces of autografts harvested from the iliac crest were trimmed according to the size and shape of the bony defects to reconstruct the medial and lateral columns, respectively. The cortical bone was directed outward, and the cancellous side was directed inward. The total bone loss was estimated by measuring the humeral length. Then, K-wires were inserted for temporary fixation.

Finally, to optimize the stability of the bony structure, the distal humerus was stabilized using anatomical locking compression plates via a parallel configuration (Zimmer Biomet, USA). Several K-wires were left for the fixation of the tiny fragments.

After internal fixation, the elbow joint exhibited almost full range of motion during passive flexion and extension (see Fig. 3). The remaining iliac crest autografts and bone callus were cut into several strip-shaped bone chips and implanted around the supracondylar level.

Finally, the olecranon osteotomy site was reduced and fixed by tension band wires. We performed subcutaneous transposition of the ulnar nerve using soft tissue sling to prevent direct contact and irritation from the hardware. The muscles and deep fascia were sutured carefully to cover the bone grafts and internal fixation site. The donor site was closed by direct suturing.

**Fig. 4:**
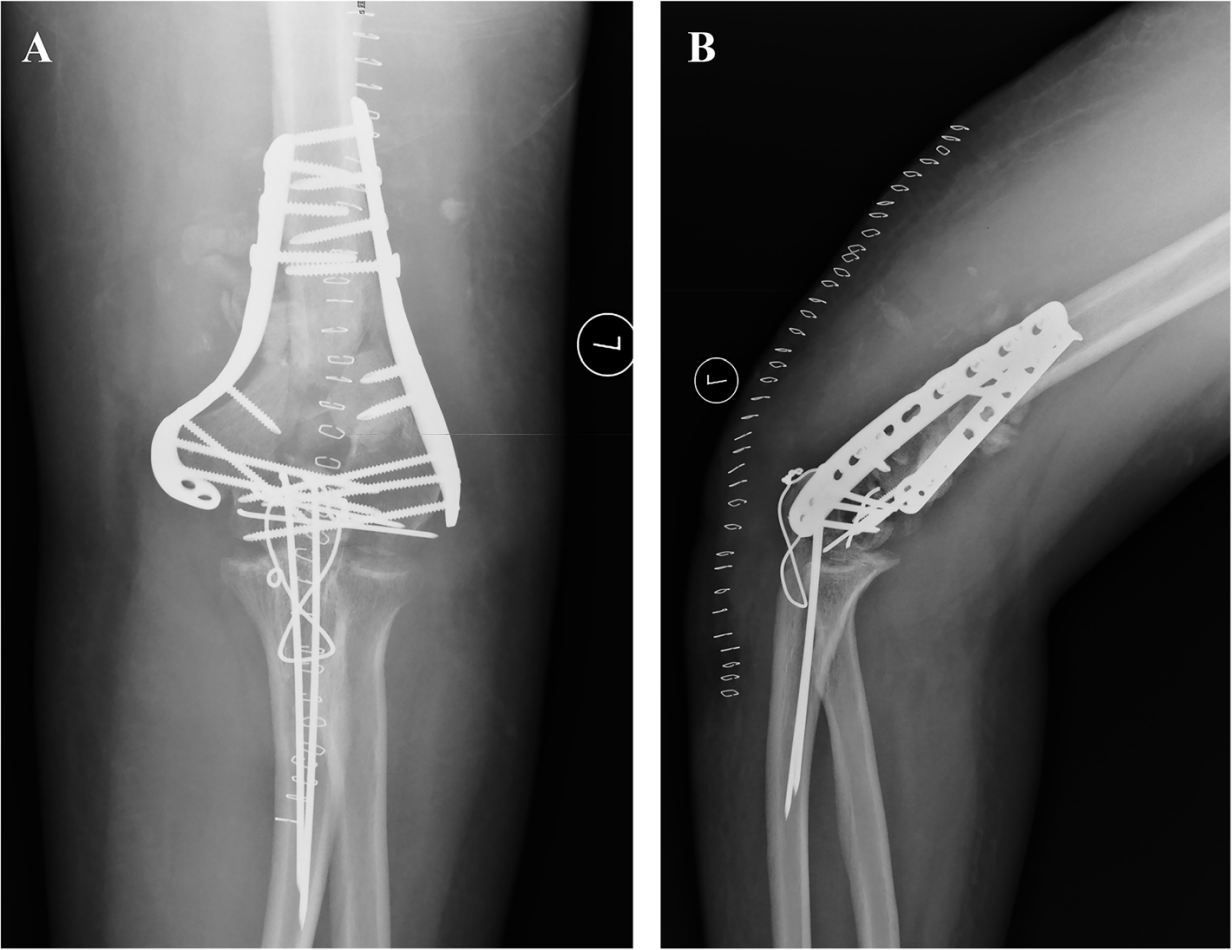
Postoperative AP (**a**) and lateral (**b**) view of X-rays after fixation

After the surgery, standard AP and lateral radiographs of the elbow joint were taken to evaluate the quality of reconstruction (see Fig. 4).

The drainage tube was removed 24 h after surgery. Active exercises of the hand and wrist, isometric contractions of the biceps and forearm muscles, and active elbow flexion and extension exercises were initiated on the second day after surgery.

### Follow-up results

Routine follow-ups were carried out. The fracture healed at 3 months postoperatively, and the radiographs showed the presence of a continuous callus passing through the fracture line. Six months after the index surgery, the patient had a painless elbow joint and almost full recovery (125° elbow flexion and 0° extension, 90° forearm supination and 65° pronation). The Mayo elbow performance score (MEPS) was 100 (excellent).

Three years after the index surgery, the patient came to our department for hardware removal due to psychological factors. He was pain free at the affected elbow joint. The flexion-extension range of motion was 130–0°, and the supination-pronation range of rotation was 90–80°. The MEPS was 100 points. The patients was very satisfied.

**Fig. 5:**
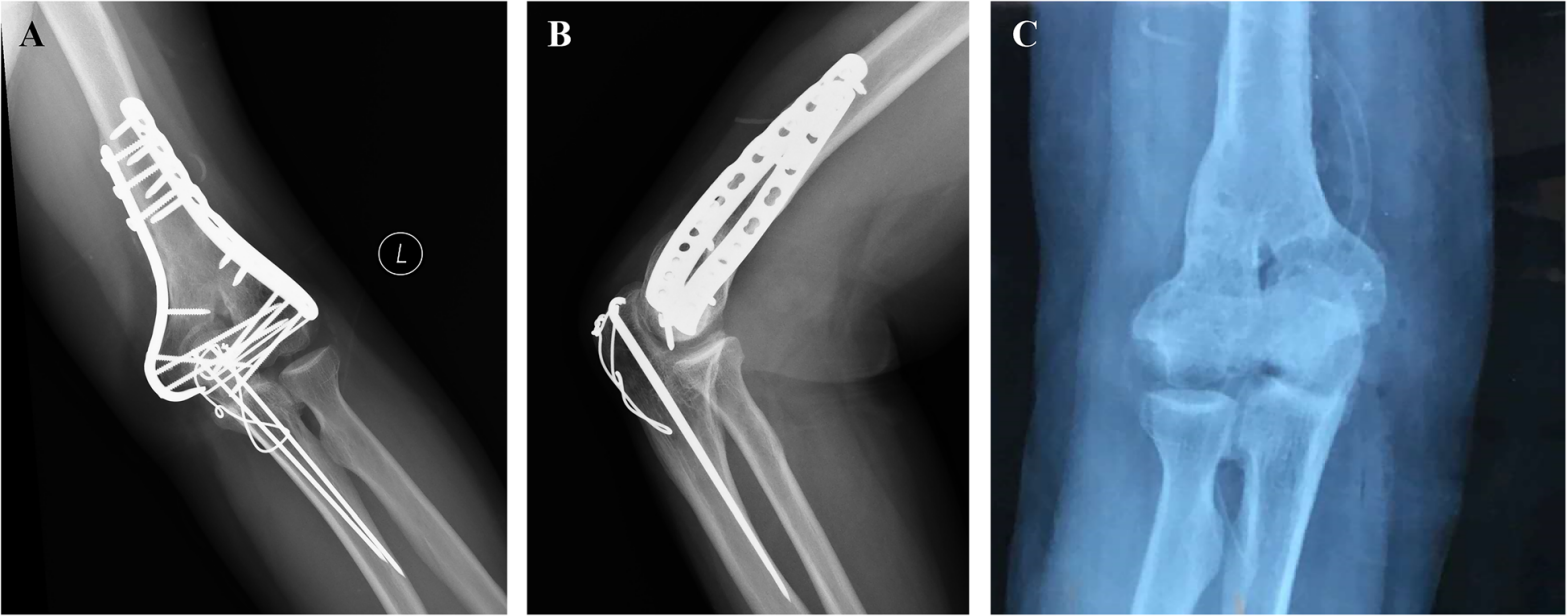
Postoperative AP (**a**) and lateral (**b**) view of X-rays after 3 years, and postoperative radiograph after hardware removal (**c**)

Secondary displacement or the loss of reduction, implant loosening or internal fixation breakage, and obvious articular degeneration were not observed. No other postoperative complications, such as infection, nonunion, delayed union, ulnar nerve symptoms, or donor site pain, occurred after the initial internal fixation procedure. After hardware removal, the overall bony structure of the affected elbow joint remained intact with only a partial deformity at the lateral column, which had no significant influence on the overall functional outcome (see Fig. 5).

## Discussion and conclusion

Distal humeral fractures with massive bone defects are challenging for orthopedic surgeons to treat, especially with open fractures and chronic intra-articular involvement. For elderly patients, such cases are not uncommon due to osteoporosis, and total elbow arthroplasty is widely used and has been shown to lead to satisfactory prognoses in previous literature [6]. However, these cases are rarely seen in young and active patients with adequate bone stock unless they suffer from extremely high-energy trauma. In such cases, TEA is usually not viable owing to its tremendous influence on patients’ upper limb strength, which is essential for them to participate in daily activities and moderate- to high-intensity physical exercises [9].

In this case report, we treated a young and active patient with such a devastating situation by arthrolysis, open reduction, internal fixation via a parallel configuration, and iliac crest autografting. We successfully reconstructed the triangular structure of the distal humerus, and the patient achieved excellent range of motion and an excellent functional score, without minor or major postoperative complications. We believe that rigid internal fixation (especially parallel double-plate configuration), the appropriate shape and position of the autografts, adequate reduction, and early range of motion exercises are crucial for achieving satisfactory clinical outcomes. However, there are still some controversies in the management of such cases.

### Approach

Numerous approaches for distal humeral fractures have been described [1]. All these methods involve a posterior skin incision and various strategies of working through or around the triceps muscle, including the paratricipital (Alonso-Llames), triceps-reflecting (Bryan-Morrey) [10], triceps-reflecting anconeus pedicle (TRAP), triceps-splitting, and olecranon osteotomy approaches [1]. However, for chronic cases with comminuted articular surfaces, adequate surgical exposure is essential and necessary for anatomic reduction as well as successful internal fixation [11]. Therefore, we chose the chevron olecranon osteotomy approach and fixed the osteotomy site with tension band wires instead of plates to avoid major complications such as deep infection and the need for revision surgery [12].

### Internal fixation configuration

For distal humeral fractures, parallel and orthogonal double plating techniques are most commonly and widely applied in clinical practice and biomechanical studies [1]. Most clinical cohort studies comparing these two configurations did not show statistically significant differences in the functional outcomes or postoperative complications [13, 14]. However, regarding the biomechanical properties, the parallel double-plate configuration demonstrated significantly higher stiffness than the orthogonal double-plate configuration in different settings, including axial, bending or torsional experiments [15, 16]. Therefore, for the case in our study, which was associated with severe comminution and massive bone defects in the articular surface and both columns, we preferred the parallel double-plate configuration, which forms an arch-like structure to strengthen the stability of the distal humeral triangle to the greatest extent possible.

### Structural iliac crest autograft

Several methods have been used to address nonunion and bone defects in chronic cases, including vascularized fibular grafting, autologous iliac crest bone grafting, and allografting [17–19]. Iliac crest bone grafts contain both cancellous and cortical bone surfaces and are relatively easy and convenient to harvest. During surgery, iliac crest bone grafts can be customized into desired sizes and shapes and fit into bone defects. We recommend the use of such autografts, which facilitate optimal anatomic reconstruction of the triangular structure of the distal humerus. Comminution in the central area of the articular surface requires structural bone grafts, whose lower margin should not reach the cartilage of the olecranon to prevent secondary joint-space narrowing, incongruence, and even posttraumatic osteoarthritis.

### Metaphyseal shortening

Bone defects at the supracondylar level may be managed successfully with humeral shortening within a certain length. A study showed that humeral shortening with no more than 2 cm has no significant impact on the kinematic and biomechanical properties of the elbow joint [20]. After humeral shortening and adequate autograft implantation, there will be enough compression between fracture fragments, thus lowering the risk of nonunion or delayed union. Nevertheless, the olecranon and coronoid fossa must be reconstructed to facilitate the recovery of elbow flexion and extension.

### Ulnar nerve

Subcutaneous transposition of the ulnar nerve has been commonly applied in treating distal humeral fractures. This procedure can provide good soft tissue protection for the ulnar nerve and prevent irritation of the hardware. However, during this procedure, devascularization after excessive stripping and stretching of the ulnar nerve may cause postoperative neural deficits [21]. Therefore, the management of the ulnar nerve remains controversial. Ruan et al. [22] prospectively evaluated the efficacy of ulnar nerve transposition in patients with preoperative ulnar nerve symptoms and found that the patients with ulnar nerve transposition exhibited significantly better neural recovery than the patients without transposition. However, for patients without preoperative neural deficits, Vasquez et al. [23] found that there is no significant difference in the incidence of postoperative ulnar nerve symptoms between these two procedures. In general, it is recommended to select the treatment for the ulnar nerve based on the preoperative symptoms, intraoperative conditions, and experiences of the surgeons. Here, we preferred subcutaneous transposition to avoid irritation of the autografts and implants, especially the medial plate.

To summarize, for complex chronic distal humeral fractures in young and active patients, especially when massive bone defects and severe articular comminution are concomitant, we proposed a set of standardized operation protocol for the reconstruction of the distal humerus using metaphyseal shortening and structural iliac crest bone autografting together with open reduction and internal fixation via a parallel double-plate configuration, which have led to satisfactory clinical outcomes in our case. However, studies with a longer follow-up period are still needed to further evaluate long-term clinical prognoses, especially regarding the development of posttraumatic osteoarthritis, which is more prone to occur in our patient, whose articular surfaces have been reconstructed by iliac crest autografts.

## Data Availability

The datasets used and/or analysed during the current study are available from the corresponding author on reasonable request.

## Abbreviations

TEA: Total Elbow Arthroplasty
CRF: C-reactive Protein
ESR: Erythrocyte Sedimentation Rate
AP: Anteroposterior
AO/ASIF: Association for Osteosynthesis/ Association for the Study of Internal Fixation
MEPS: Mayo Elbow Performance Score
